# Some Human Lessons in War-Time

**Published:** 1916-11-04

**Authors:** 


### LIFE AND DEATH.

### Some Human Lessons in War-Time.

### VI.—WHEN LIFE IS AND IS NOT A FAILURE.*

UNLESS a man or woman realises that individually he is responsible as a trustee to God for his own life, he may fail to live, and merely exist. Life is not to live, but to be in health, to be well in spirit, to be well in body, to possess a sound mind working with a sound physical system. There are relatively few who realise the reality of this trusteeship in their own case and continuously endeavour to live up to it throughout life. It is rather remarkable that observation of the character and nature of the teaching and sermons from the pulpits of all denominations leads to the conclusion that the cleric seldom realises each man's trusteeship to Almighty God for his own life. Practical religion, much less personal religion, is seldom taught from the pulpit; that is why the state of the Church, not only in Great Britain, but throughout the English-speaking world, has largely failed to set up and maintain a high standard of personal religion. Since the war the interest of all classes has gradually awakened to the importance of religion, but this general interest in the many is quite consistent with a low standard of religious attainment in individuals; that is, low in comparison with what might be expected from the motive power that the Gospel brings to bear upon the heart.

### THE PERSISTENCE OF A LOW STANDARD.

This state of affairs is unfortunately not merely of recent date. An eminent divine, the late Dean Goulborn, of Norwich Cathedral, about fifty years ago, in lamenting this low standard, properly insisted that to many questions respecting man's moral condition we can perhaps give a satisfactory answer. If it is asked where is integrity, where is amiability, where is social worth, where is attendance upon the ordinances of religion, where are alms, deeds, and charitable institutions, many instances can be produced, but be it remembered that many, if not all, of these fruits can be borne by unregenerate human nature. The annals of heathenism record numerous instances of integrity and even ascetic self-denial among the philosophers and many others of high moral tone, and brilliant disinterestedness among the people at large; but life must be a failure for every man and woman where Christian saintliness does not go beyond this as being the product of much higher agencies. Is it true that, though the public may be religious as a public, in individuals the salt has lost its savour? Most people can speak volubly upon controversial subjects, but where are the men and women upon whose hearts truth, which is at stake in the controversy, is making every day, by means of prayer and meditation, a deeper imprint? In the case of the laity, every man or woman who realises a responsibility to God as a trustee for his own life is daily moulding himself nearer to the Divine idea of Perfect Man already to us revealed.

In the great majority, however, the call to arouse themselves to the apprehension of their spiritual nature is imperative, and of vital importance. What have the Churches, their clergy and ministers done to awaken the majority of men and women, who at the present time do not live in any real meaning of the word, to shed the isolation of mere existence? Such individuals have no sense of their trusteeship to God and no realisation of their spiritual nature and its claims for continuous development and cultivation. Some there are no doubt, and we hope many, in the ranks of the clergy and ministers of all denominations, who realise the importance of the spiritual nature of man and the claim it has upon them to feed the people and promote its vigorous and continuous growth. But what of the others, the men who fear to be outspoken and vigorous teachers of the truth, and to impress the truth insistently from their pulpits and everywhere upon the men and women who come within their sphere of influence and earnest inquiry into the conditions which prevail, and which have moved those responsible to organise a great Mission of awakement amongst English-speaking people. At the present, time has naturally directed the eyes of the inquirers in the first place to the Christian ministry as now exercised; indeed, the entering by the Bishops and leaders of religious thought into this Mission of awakement is direct evidence that in their view the results of the Gospel at present are not what they should be, and that it is fair to conclude that there are defects in the instrumentality employed. The ministry of God's word is a great appointed means for the perfecting of the saintly or Christ-like character in man. Is there, then, any flaw in this ministry which may account for the low standard of personal religion of which we have spoken?

### POLITICS IN THE PULPIT.

In previous articles we have dealt with some aspects of this question, and to-day it necessarily crops up again out of the inquiry when life is, and is not, a failure. The laity, and especially the men of the laity, have increasingly of late been cut to the quick by the perfunctory, conventional, inanimate, and sleepy attitude of certain clergy and ministers, who seem to have lost their interest and deadened their apprehension in regard to the spiritual nature of men and women. Political

* Previous articles appeared on September 23 and 30 and October 7, 14, and 21.

pulpit propaganda on the one hand and lack of dynamic in the conduct of Divine service on the other seem to some onlookers as the very root of their angry contempt on occasion. It is perfectly certain that each individual who is alive to his spiritual nature will gratefully join hands with the bishops and religious teachers who are organising the great Mission now being carried out in the Metropolis, which we hope may be extended to all parts of the British Empire. It would be of supreme importance if this Mission could be extended, too, to every community in the United States of America, where there is felt real need for men upon whose soul Truth is graving a deeper impress day by day; for men whose lives are daily strengthened by prayer and meditation. This fact is impressed upon us by the knowledge that the articles which have already appeared in this series are being republished week by week in a considerable number of the leading daily newspapers of America.

EXISTENCE *versus* LIFE.

Horace contended with force and truth that unless the vessel be pure whatever you put in it will turn sour. That is why we have thought it well to consider when life is and is not a failure. The pure in heart are fully alive to their spiritual nature, whether they be bishops, clergymen, ministers, religious leaders, teachers, or lay men and women. For every one of them life is in no sense a failure. They have come to recognise the trusteeship of the life which has been conferred upon them. Each and all have long ceased merely to exist, and have come to live with ever-increasing vitality owing to the subordination of their physical to their spiritual nature. When a man or woman has reached this stage of development and entered heart and soul upon the work of a trustee for his own life, wisdom takes the helm, and his future progress, especially where he has the uplifting influence and the constant co-operation of a wise and awakened minister of Christ, should be inspired and blessed. The vessel will then be pure, and whatever you put into it will never turn sour. The policy of those whose lives are not a failure, as we have often known, is to exert their energies to get wisdom. They habitually study and read continuously two books, the Bible and history—the history of their nation—for nationality is the soul of human life. The Bible is not only the most wonderful but the most instructive and enlightening book which the world has ever possessed. The constant perusal of its pages enables the intelligent reader as years advance to realise increasingly how accurate and all-inclusive is the Bible in regard to the conduct, failings, habits, needs, and development of men and women. God's dealings with man are there set forth clearly, and are illustrated by examples which soon bring comfort, conviction, and strength to the habitual reader of the Scriptures. Thus strengthened and encouraged, both men and women may develop their spiritual nature to the state where self is subordinated. Then virtue, which is the only and true nobility, speedily dominates the individual who, having shed isolation in mere existence, has entered upon life. Health and blessedness follow with a new sense of purity and of man's and woman's responsibility to God that then comes to them and sustains them throughout their life on earth.

